# Global survey on the surgical management of patients affected by colorectal cancer with synchronous liver metastases: impact of surgical specialty and geographic region

**DOI:** 10.1007/s00464-023-09917-8

**Published:** 2023-03-06

**Authors:** Jasper Paul Sijberden, Antonino Spinelli, Alessandro Ferrero, Manish Chand, Steven Wexner, Marc G. Besselink, Ibrahim Dagher, Giuseppe Zimmitti, Burak Görgec, Antonio de Lacy, Mayank Roy, Pieter Tanis, Carlo Tonti, Mohammed Abu Hilal

**Affiliations:** 1 Department of Surgery, Fondazione Poliambulanza Istituto Ospedaliero, Via Leonida Bissolati 57, Brescia, Italy; 2 Department of Surgery, Amsterdam UMC Location University of Amsterdam, Amsterdam, The Netherlands; 3 Cancer Center Amsterdam, Amsterdam, The Netherlands; 4 Department of Biomedical Sciences, Humanitas University, Pieve Emanuele, Milan, Italy; 5 IRCCS Humanitas Research Hospital, Rozzano, Milan, Italy; 6 Department of General and Oncological Surgery, Ospedale Mauriziano, Turin, Italy; 7 Wellcome EPSRC Centre for Interventional and Surgical Sciences (WEISS), University College London, London, UK; 8 Department of Colorectal Surgery, Cleveland Clinic Florida, Weston, Florida USA; 9 Department of Digestive Minimally Invasive Surgery, Antoine Béclère Hospital, Paris, France; 10 Gastrointestinal Surgery, Institut Clínic de Malaties Digestives I Metabòliques, Hospital Clínic, University of Barcelona, Barcelona, Spain; 11 Department of General Surgery, Cleveland Clinic Florida, Weston, Florida USA; 12 Department of Oncological and Gastrointestinal Surgery, Erasmus MC Cancer Institute, Rotterdam, The Netherlands; 13 Department of Surgery, University Hospital Southampton NHS Foundation Trust, Southampton, UK

**Keywords:** Colorectal cancer, Synchronous colorectal liver metastases, Surgical procedures, Clinical practice pattern, Survey

## Abstract

**Background:**

Consensus on the best surgical strategy for the management of synchronous colorectal liver metastases (sCRLM) has not been achieved. This study aimed to assess the attitudes of surgeons involved in the treatment of sCRLM.

**Methods:**

Surveys designed for colorectal, hepato-pancreato-biliary (HPB), and general surgeons were disseminated through representative societies. Subgroup analyses were performed to compare responses between specialties and continents.

**Results:**

Overall, 270 surgeons (57 colorectal, 100 HPB and 113 general surgeons) responded. Specialist surgeons more frequently utilized minimally invasive surgery (MIS) than general surgeons for colon (94.8% vs. 71.7%, *p* < 0.001), rectal (91.2% vs. 64.6%, *p* < 0.001), and liver resections (53% vs. 34.5%, *p* = 0.005). In patients with an asymptomatic primary, the liver-first two-stage approach was preferred in most respondents’ centres (59.3%), while the colorectal-first approach was preferred in Oceania (83.3%) and Asia (63.4%). A substantial proportion of the respondents (72.6%) had personal experience with minimally invasive simultaneous resections, and an expanding role for this procedure was foreseen (92.6%), while more evidence was desired (89.6%). Respondents were more reluctant to combine a hepatectomy with low anterior (76.3%) and abdominoperineal resections (73.3%), compared to right (94.4%) and left hemicolectomies (90.7%). Colorectal surgeons were less inclined to combine right or left hemicolectomies with a major hepatectomy than HPB and general surgeons (right: 22.8% vs. 50% and 44.2%, *p* = 0.008; left: 14% vs. 34% and 35.4%, *p* = 0.002, respectively).

**Conclusion:**

The clinical practices and viewpoints on the management of sCRLM differ between continents, and between and within surgical specialties. However, there appears to be consensus on a growing role for MIS and a need for evidence-based input.

**Graphical abstract:**

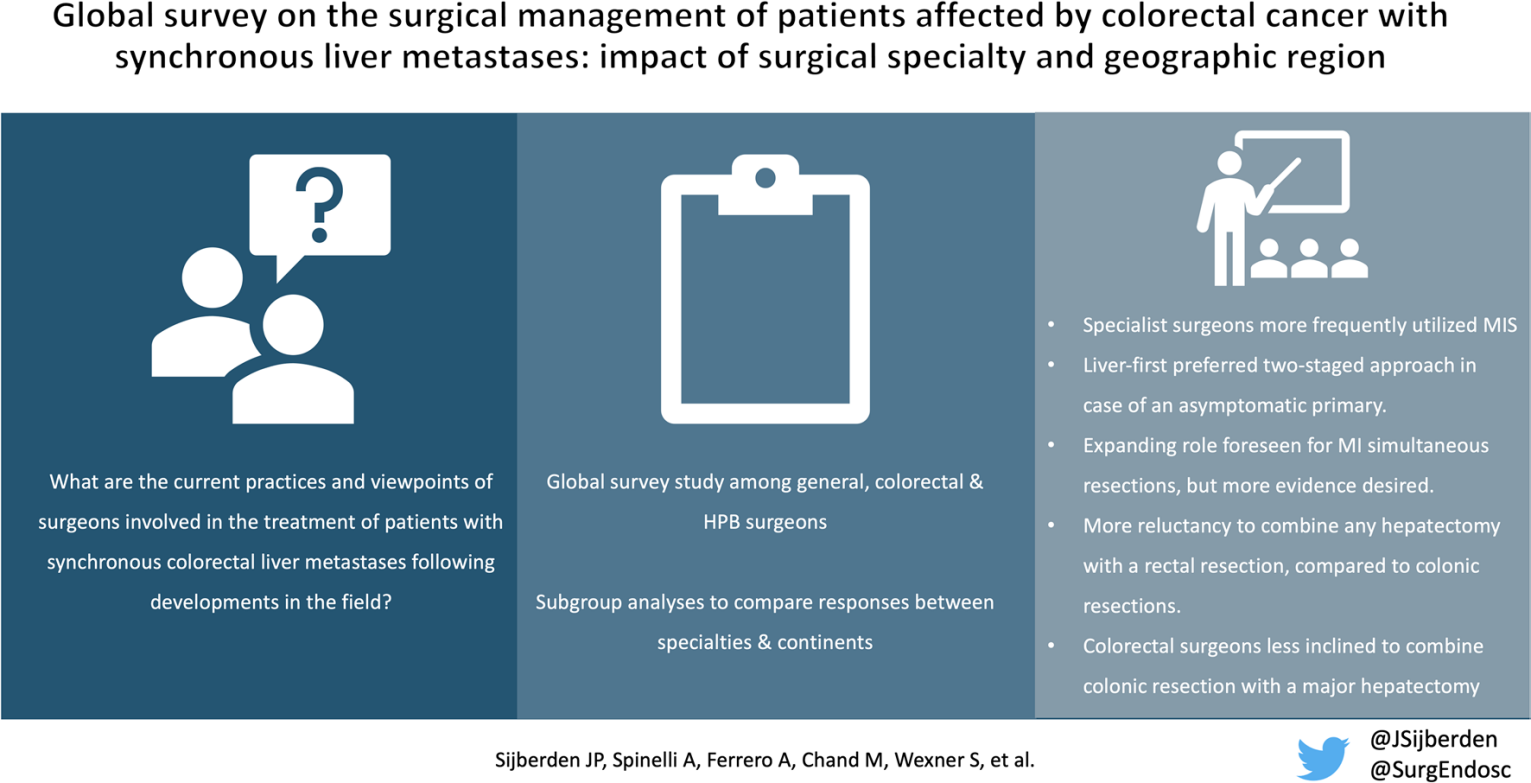

**Supplementary Information:**

The online version contains supplementary material available at 10.1007/s00464-023-09917-8.

Synchronous colorectal liver metastases (sCRLM) are present in approximately 13.5% of newly diagnosed patients with colorectal cancer (CRC), and in a substantial proportion of these patients metastatic disease is confined to the liver [1, 2]. Despite significant improvements in the oncological and surgical treatment of patients with colorectal liver metastases (CRLM), the management of these patients remains challenging and the optimal treatment strategy for each individual patient remains to be defined [3]. An illustrative example of this can be found in a recent study wherein 43 expert liver surgeons disclosed their preferred treatment strategies for 10 patients with CRLM (8 of whom had sCRLM). In this study, a strikingly low degree of agreement among the participating surgeons was observed, with a Cohen’s kappa ranging from 0 to 0.39 in more than half of the cases reviewed [4].

In patients with resectable disease, the timing of resection of the primary CRC and CRLM is perhaps the most debated subject. The traditional surgical strategy for these patients has been the “classical” two-stage resection wherein the primary CRC is resected first followed by a liver resection [5]. It has been considered that the interval between the two surgical procedures allows the identification of malignancies with aggressive biology, enabling the selection of patients who will likely benefit from a second major surgical procedure. An alternative to the traditional approach is the “reverse” or liver-first approach, involving a two-stage resection wherein the liver resection is performed first [6]. It has been suggested that a delayed resection of the liver metastases may lead to un-resectability, which is rarely the case for the primary tumour. Furthermore, complications after resection of the primary tumour may delay chemotherapy or the second surgical procedure. In recent years, due to increasing surgical experience and improvements in peri-operative management, simultaneous resections of both the primary CRC and CRLM were proposed as an effective novel surgical intervention for patients with sCRLM [7]. Simultaneous resections have been associated with a shorter length of hospital stay (LOS) and non-inferior survival, morbidity and mortality rates when compared with the two-stage procedure. [7–9]

For all three strategies, both open and minimally invasive surgery (MIS) have been shown to be feasible, safe and oncologically efficient [9–13]. However, to date, it remains unclear which strategy and approach should be preferred in patients with sCRLM. Previous studies have reported conflicting outcomes in this highly heterogeneous patient population and current guidelines mainly state that treatment plans should be personalized and based on the expertise of a multidisciplinary team (MDT) [8, 9, 14, 15]. In this day and age, no study has investigated surgeons’ attitudes towards the management of patients with sCRLM on a global scale. Therefore, the aim of this survey study is to assess the current practices and viewpoints of surgeons involved in the treatment of patients with sCRLM, with a special focus on possible cross-continental and cross-specialty differences.

## Methods

### Survey design

Three different versions of a survey, to be presented to fully licensed colorectal, hepato-pancreato-biliary (HPB), and general surgeons (defined as surgeons performing both colorectal and liver resections in patients affected by sCRLM), were developed by three of the authors (BG, JS and MAH). Thereafter, an international steering committee consisting of colorectal and HPB surgeons (MB, MC, ID, AF, AdL, MR, AS, PT, GZ, CT and SW), several of whom had experience in conducting survey research, assessed the surveys’ comprehensibility and face validity. Final adjustments were made and an online version of the survey was developed using Google Forms® (Google, Mountain View, CA, USA). Several multinational societies, known for their large following and active membership, namely the International Hepato-Pancreato Biliary Association (IHPBA), the European Society of Coloproctology (ESCP), the American Society of Colon and Rectal Surgeons (ASCRS), the Society of American Gastrointestinal and Endoscopic Surgeons (SAGES), and the European Association of Endoscopy Surgery (EAES) were asked to support this project. The IHPBA, SAGES and ESCP agreed and disseminated the survey, whereafter it was available for completion between February 12, 2021, and May 12, 2021. Additionally, the survey was promoted on social media. Respondents were asked to register their name and institution to prevent double entries. Furthermore, respondents were asked to clarify if they, within the scope of the survey, only performed colorectal resections, liver resections or both after which they were taken through the corresponding sections of the survey. The survey contained 36 to 44 questions (depending on the surgical specialty) covering several domains, including the working relationship between surgical specialties, adopted surgical approaches, surgical management of patients with sCRLM and opinions on outcomes after simultaneous resections. Submitting the survey was only possible after answering all questions. The survey is reported in the *supplementary materials*.

### Statistical analysis

Categorical data are reported as frequencies and percentages. Continuous normally distributed data are reported as mean with standard deviation (SD), non-normally distributed data as median with interquartile range (IQR). Normality was checked by visually inspecting histograms and Q–Q plots. Subgroup analyses were performed to compare viewpoints and used strategies between surgical specialties, practice setting (academic versus non-academic), and continents using Chi-Squared tests and Fisher’s exact test. For statistical analysis, IBM SPSS Statistics® version 27.0 (IBM, Armonk, New York, USA) was used. A two-tailed *P *value ≤ 0.05 was considered significant.

## Results

A total of 270 surgeons (57 colorectal, 100 HPB and 113 general surgeons) from 61 countries responded, with a median experience of 13 years as an attending surgeon (IQR 8–21). In the respondents’ institutions, the median annual institutional volumes were 150 CRC resections (IQR 80–200) and 60 liver resections (IQR 33.25–100), with a personal median annual volume of 32 (IQR 20–57.5) and 28.5 (IQR 15–50) resections, respectively. Further characteristics and the number of respondents per country are shown in *Table **1* and supplementary Fig. 1.

**Table 1:** Characteristics of respondents

Characteristics	*n* = 270
	*n* (%)/Median (IQR)
Scope of current clinical practice	
Colorectal surgery	57 (21.1)
Also performed liver resections earlier	22 (38.6)
Years stopped performing liver resections	4 (3–11.25)
Hepato-pancreato-biliary surgery	100 (37)
Also performed CR resections earlier	72 (72)
Years stopped performing CR resections	5 (3–10)
Both colorectal and liver surgery	113 (41.9)
Type of hospital	
Academic	179 (66.3)
Non-Academic teaching	72 (26.7)
Non-teaching	19 (7)
Years of experience as an attending surgeon	13 (8–21)
Annual institutional volume	
Colorectal cancer resections	150 (80–200)
Liver resections	60 (35.25–100)
Annual personal volume	
Colorectal cancer resections	32 (20–57.5)
Liver resections	28.5 (15–50)
Open simultaneous resection total	20 (10–50)
Open simultaneous resection annually	5 (2–10)
MI simultaneous resection total	8 (4–20)
MI simultaneous resection annually	3 (1–5)

*CR* colorectal, *MI* minimally invasive

### Organization of care for patients with sCRLM and opinions on clinical judgement

This survey confirms that the clinical decision-making process for these patients predominantly occurs in a multidisciplinary setting, as an MDT meeting was available to 94.8% of all respondents. (*Table **2*) However, respondents from Asia less often had access to an MDT (85.7% versus 94.4–100%, *p* = 0.023) (*Table **3*). Additionally, the type of MDT where patients with CRLMs were discussed differed significantly according to the hospital setting, as a shared MDT meeting (both colorectal and HPB surgery present) was more common in non-academic hospitals (57.5% vs. 33.1%, *p* = 0.003) (Supplementary table 1).

**Table 2:** Organization of care, opinions on clinical judgement and surgical strategies for patients with CRLM overall & stratified by surgical specialty

Characteristics	Overall (*n* = 270)	CR surgery (*n* = 57)	HPB surgery (n = 100)	Gen. surgery (*n* = 113)	*P *value
	*n* (%)	*n* (%)	*n* (%)	*n* (%)	
*Organization of care for patients with CRLM*					
Access to MDT meeting	256 (94.8)	54 (94.7)	95 (95)	107 (94.7)	0.994
Type of MDT					0.868
Colorectal	19 (7.4)	4 (7.4)	7 (7.4)	8 (7.5)	
HPB	34 (13.3)	4 (7.4)	19 (20)	11 (10.3)	
Shared	106 (41.4)	29 (53.7)	28 (29.5)	49 (45.8)	
Both	97 (37.9)	17 (31.5)	41 (43.2)	39 (36.4)	
Discuss cases with respective colleague before the MDT (***n* = 188**)		*n* = 21*	*n* = 60*	*n* = 107*	0.440
< 50% of cases	63 (33.5)	9 (42.9)	18 (30)	36 (33.6)	
50–99% of cases	41 (21.8)	6 (28.6)	13 (21.7)	22 (20.6)	
100% of cases	84 (44.7)	6 (28.6)	29 (48.3)	49 (45.8)	
After MDT plan overall surgical management with respective colleague(s) (***n* = 256**)	220 (85.9)	53 (98.1)	91 (95.8)	76 (71)	< 0.001
No access, but will discuss cases with respective colleague(s) (***n* = 14**)					0.660
< 50% of cases	8 (57.1)	2 (66.7)	3 (60)	3 (50)	
50–99% of cases	3 (21.4)	1 (33.3)	/	2 (33.3)	
100% of cases	3 (21.4)	/	2 (40)	1 (16.7)	
*Working relationship with respective colleagues*					0.543
Excellent	171 (63.3)	32 (56.1)	77 (77)	62 (54.9)	
Good	65 (24.1)	16 (28.1)	18 (18)	31 (27.4)	
Adequate	19 (7)	3 (5.3)	4 (4)	12 (10.6)	
Fair	4 (1.5)	2 (3.5)	/	2 (1.8)	
Poor	3 (1.1)	1 (1.8)	1 (1)	1 (0.9)	
No respective colleague in institution	8 (3)	3 (5.3)	/	5 (4.4)	
*Opinions on clinical judgement*					
Able to determine eligibility for local treatment?					0.944
No	8 (3)	3 (5.3)	3 (3)	2 (1.8)	
Solely	49 (18.1)	2 (3.5)	22 (22)	25 (22.1)	
I n context of MDT	179 (66.3)	48 (84.2)	63 (63)	68 (60.2)	
Together with a radiologist	34 (12.6)	4 (7)	12 (12)	18 (15.9)	
HPB surgeons best suited to determine eligibility for local treatment?					0.290
Yes	253 (93.7)	55 (96.5)	94 (94)	104 (92)	
Only in context of MDT	7 (2.6)	1 (1.8)	2 (2)	4 (3.5)	
Others are best suited	10 (3.7)	1 (1.8)	4 (4)	5 (4.4)	
*Surgical strategies*					
Two-stage procedure in patient with asymptomatic primary					0.542
Colorectal first	110 (40.7)	26 (45.6)	42 (42)	42 (37.2)	
Liver-first	160 (59.3)	31 (54.4)	58 (58)	71 (62.8)	
Usage of ICG fluorescence					
Colorectal resections (***n* = 168**)§	64 (38.1)	23 (40.4)		41 (36.9)	0.666
To assess vascularization	63 (37.5)	23 (40.4)		40 (36)	
For guidance during lymphadenectomies	8 (4.8)	1 (1.8)		7 (6.3)	
Liver resections (***n = *213**)	68 (31.9)		33 (33)	35 (31)	0.752
For anatomical demarcation	53 (24.9)		28 (28)	25 (22.1)	
To identify tumour borders	37 (17.4)		17 (17)	20 (17.7)	
To identify occult metastases	28 (13.1)		10 (10)	18 (15.9)	
Participated in a MI simultaneous resection	196 (72.6)	33 (57.9)	74 (74)	89 (78.8)	0.015
*Strategies for simultaneous resection*					
In my institution, MI simultaneous resection is performed by:					< 0.001
Not performed yet	53 (19.6)	15 (26.3)	12 (12)	26 (23)	
Colorectal surgeon & HPB surgeon together	155 (57.4)	33 (57.9)	84 (84)	38 (33.6)	
HPB surgeon with experience in colorectal resections	35 (13)	5 (8.8)	3 (3)	27 (23.9)	
Colorectal surgeon with experience in liver resections	27 (10)	4 (7)	1 (1)	22 (19.5)	
Number of surgeons performing MI combined resection in institution					0.062
Zero	54 (20)	16 (28.1)	12 (12)	26 (23)	
One	41 (15.2)	7 (12.3)	14 (14)	20 (17.7)	
Two	76 (28.1)	10 (17.5)	30 (30)	36 (31.9)	
Three	56 (20.7)	11 (19.3)	20 (20)	25 (22.1)	
Four or more	43 (15.9)	13 (22.8)	24 (24)	6 (5.3)	

*CRLM* colorectal liver metastases, *MDT* multidisciplinary team, *CR* colorectal, *HPB* hepato-pancreato-biliary, *ICG* indocyanine green, *MI* minimally invasive

*For colorectal specialists, this question was only asked when the “colorectal” or “both” type of MDT was chosen, for HPB specialists when the “HPB” or “both” type of MDT was chosen

§Data from 2 respondents missing

**Table 3:** Organization of care, opinions on clinical judgement and surgical strategies for patients with CRLM per continent

*Characteristics*	North America (*n* = 34)	South America (*n* = 18)	Europe (*n* = 148)	Africa (*n* = 8)	Asia (*n* = 56)	Oceania (*n* = 6)	*P *value
	*n* (%)	*n* (%)	*n* (%)	*n* (%)	*n* (%)	*n* (%)	
Scope of current clinical practice							< 0.001
Colorectal surgery	2 (5.9)	1 (5.6)	41 (27.7)	1 (12.5)	10 (17.9)	2 (33.3)	
HPB surgery	21 (61.8)	14 (77.8)	42 (28.4)	1 (12.5)	18 (32.1)	4 (66.7)	
Colorectal and liver surgery	11 (32.4)	3 (16.7)	65 (43.9)	6 (75)	28 (50)	/	
*Organization of care for patients with CRLM*							
Access to MDT meeting	34 (100)	17 (94.4)	143 (96.6)	8 (100)	48 (85.7)	6 (100)	0.023
Type of MDT							0.764
Colorectal	4 (11.8)	1 (5.9)	8 (5.6)	/	6 (12.5)	/	
HPB	9 (26.5)	/	20 (14)	1 (12.5)	3 (6.3)	1 (16.7)	
Shared	6 (17.6)	10 (58.8)	60 (42)	4 (50)	25 (52.1)	1 (16.7)	
Both	15 (44.1)	6 (35.3)	55 (38.5)	3 (37.5)	14 (29.2)	4 (66.7)	
Discuss cases with respective colleague before the MDT (***n* = 188**)*							0.360
< 50% of cases	8 (27.6)	/	42 (38.5)	/	11 (35.5)	2 (40)	
50–99% of cases	7 (24.1)	1 (12.5)	23 (21.1)	2 (33.3)	6 (19.4)	2 (40)	
100% of cases	14 (48.3)	7 (87.5)	44 (40.4)	4 (66.7)	14 (45.2)	1 (20)	
After MDT plan overall surgical management with respective colleague(s) (***n* = 256**)	31 (91.2)	14 (82.4)	122 (85.3)	6 (75)	41 (85.4)	6 (100)	0.733
No access, but will discuss cases with respective colleague(s) (***n* = 14**)							0.208
< 50% of cases		1 (100)	3 (60)		4 (50)		
50–99% of cases		/	2 (40)		1 (12.5)		
100% of cases		/	/		3 (37.5)		
*Working relationship with respective colleagues*							0.314
Excellent	26 (76.5)	13 (72.2)	90 (60.8)	6 (75)	31 (55.4)	5 (83.3)	
Good	7 (20.6)	3 (16.7)	39 (26.4)	1 (12.5)	14 (25)	1 (16.7)	
Adequate	1 (2.9)	/	9 (6.1)	/	9 (16.1)	/	
Fair	/	/	2 (1.4)	/	2 (3.6)	/	
Poor	/	1 (5.6)	2 (1.4)	/	/	/	
No respective colleague in institution	/	1 (5.6)	6 (4.1)	1 (12.5)	/	/	
*Opinions on clinical judgement*							
Able to determine eligibility for local treatment?							0.072
No	/	1 (5.6)	6 (4.1)	/	1 (1.8)	/	
Solely	8 (23.5)	1 (5.6)	29 (19.6)	2 (25)	9 (16.1)	/	
In context of MDT	23 (67.6)	14 (77.8)	101 (68.2)	3 (37.5)	32 (57.1)	6 (100)	
Together with a radiologist	3 (8.8)	2 (11.1)	12 (8.1)	3 (37.5)	14 (25)	/	
HPB surgeons best suited to determine eligibility for local treatment?							0.296
Yes	33 (97.1)	18 (100)	137 (92.6)	7 (87.5)	53 (94.6)	5 (83.3)	
Only in context of MDT	/	/	6 (4.1)	/	1 (1.8)	/	
Others are best suited	1 (2.9)	/	5 (3.4)	1 (12.5)	2 (3.6)	1 (16.7)	
*Surgical strategies*							
Two-staged procedure in patient with asymptomatic primary							0.016
Colorectal first	12 (35.3)	7 (38.9)	52 (35.1)	2 (25)	32 (57.1)	5 (83.3)	
Liver-first	22 (64.7)	11 (61.1)	96 (64.9)	6 (75)	24 (42.9)	1 (16.7)	
Usage of ICG-fluorescence							
Colorectal resections (***n* = 168**)	8 (61.5)	/	49 (47.1)	2 (28.6)	5 (13.2)	/	0.001
Liver resections (***n* = 213**)	14 (43.8)	/	40 (37.4)	1 (14.3)	13 (28.3)	/	0.012
Participated in a MI simultaneous resection	24 (70.6)	12 (66.7)	115 (77.7)	6 (75)	36 (64.3)	3 (50)	0.323
*Strategies for simultaneous resection*							
In my institution, MI simultaneous resection is performed by:							0.867
Not performed yet	7 (20.6)	6 (33.3)	23 (15.5)	3 (37.5)	12 (21.4)	2 (33.3)	
Colorectal surgeon & HPB surgeon together	22 (64.7)	11 (61.1)	81 (54.7)	3 (37.5)	34 (60.7)	4 (66.7)	
HPB surgeon with experience in colorectal resections	4 (11.8)	1 (5.6)	23 (15.5)	/	7 (12.5)	/	
Colorectal surgeon with experience in liver resections	1 (2.9)	/	21 (14.2)	2 (25)	3 (5.4)	/	
Number of surgeons performing MI combined resection in institution							0.550
Zero	6 (17.6)	6 (33.3)	24 (16.2)	4 (50)	13 (23.2)	1 (16.7)	
One	3 (8.8)	3 (16.7)	26 (17.6)	1 (12.5)	8 (14.3)	/	
Two	11 (32.4)	5 (27.8)	37 (25)	3 (37.5)	17 (30.4)	3 (50)	
Three	7 (20.6)	2 (11.1)	39 (26.4)	/	8 (14.3)	/	
Four or more	7 (20.6)	2 (11.1)	22 (14.9)	/	10 (17.9)	2 (33.3)	

*CRLM* colorectal liver metastases, *MDT* multidisciplinary team, *MI* minimally invasive

*For colorectal specialists this question was only asked when the “colorectal” or “both” type of MDT was chosen, for HPB specialists when the “HPB” or “both” type of MDT was chosen

Despite the general agreement on the importance of patients’ discussion in an MDT, a discrepancy was noted on the tendency to plan surgical timings and strategies when resection of both the primary CRC and CRLM was advised. In fact, more than a quarter of the general surgeons would take a sole decision while HPB and colorectal surgeons tend to collaborate with another specialist (29% vs. 4.2% and 1.9%, respectively, *P* < 0.001). Surgeons who worked in a non-academic setting were also less likely to collaborate with another specialist in this regard (20.7% vs. 10.7%, *p* = 0.029) (Supplementary table 1).

Of all surgeons, 66.3% stated that they were only able to determine the eligibility of CRLM for local treatment with support of the MDT, while only 18.1% of the surgeons stated that they were able to determine this eligibility by themselves. HPB surgeons were generally considered best suited to determine the eligibility of CRLM for local treatment (93.7% of all respondents). Lastly, surgeons often (87.4%) experienced a positive working relationship with their colleagues from another surgical specialty. However, this relationship was described as adequate by 7% and poor by 2.6% of the respondents. Reasons for poor working relationships included poor communication, different views on treatment strategies and working independently of each other. (Table 2).

### Adopted surgical approaches for colon resections

Colorectal surgeons often had adequate experience in performing colon resections using an open or laparoscopic approach (83.7% and 73.7% of respondents had performed > 50 resections using these respective approaches). Adequate experience with the robotic approach was rare (3.6% of respondents with > 50 robotic resections). Laparoscopy was the preferred personal approach of most colorectal surgeons (93%), although it was not always the standard approach in colorectal surgeons’ institutions (only 31.6% declared that > 75% of the colon resections in their centre were performed laparoscopically) (Supplementary Fig. 2)*.*

**Fig. 1:**
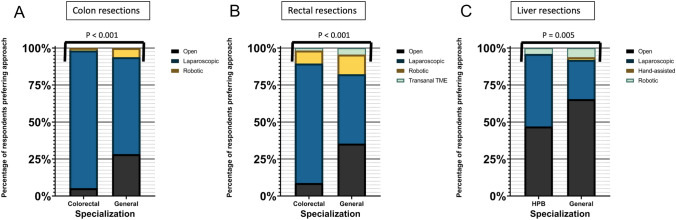
Personally preferred approaches of respondents for **A** colon resections, **B** rectal resections and **C** liver resections

Assessing the adopted approaches by general surgeons, substantial experience in performing colon resections using the open and laparoscopic approach was common among the respondents (70.8% and 51.4% of surgeons had performed > 50 resections using these respective approaches). Considerable experience with the robotic approach was also uncommon in this subgroup (7% of surgeons had performed > 50 robotic colon resections). While the laparoscopic approach was preferred by more than half of the respondents (65.5%), only 23% of the general surgeons stated that > 75% of the colon resections in their respective centre were performed using this approach (Fig. 1 & supplementary Fig. 2).

### Adopted surgical approaches for rectal resections

As expected, based on the incidence of colon and rectal cancer, a smaller proportion of the responding colorectal surgeons had considerable experience in performing rectal resections using the open or laparoscopic approach when compared to colon resections (52.6% and 42.2% with > 50 resections, respectively). Substantial experience (> 50 resections) with the robotic approach (3.6%) and Transanal total mesorectal excision (TaTME) (2.4%) were scarce. Although the laparoscopic approach was predominantly preferred for rectal resections (80.7%), its usage in colorectal subspecialists’ centres was more limited than for colon resections (19.3% stated that > 75% of the rectal resections in their centre were performed using this approach) (Supplementary Fig. 3).

Among general surgeons, 52.2% and 30% had performed > 50 rectal resections using the open and laparoscopic approach, respectively. Like in the subgroup of colorectal surgeons, only a very small proportion of the general surgeons had performed > 50 resections using the robotic approach (2.7%) and Transanal TME (1.8%). While laparoscopy was also favoured in this subgroup, the proportion of general surgeons preferring this approach (46.9%) was considerably lower than in the subgroup of colorectal surgeons (80.7%). Only in 12.4% of the general surgeons’ institutions, laparoscopy was the reference approach (Utilized in > 75% of the patients requiring a rectal resection) (Fig. 1 & supplementary Fig. 3).

### Adopted surgical approaches for liver resections

Considerable experience in performing liver resections using the open approach was most common among HPB surgeons: 73% of the respondents had performed > 50 open resections, while 40% had performed > 50 laparoscopic resections and only 3% had performed > 50 robotic resections. Although 49% of the HPB surgeons currently preferred to use the laparoscopic approach for liver resections, only 21% of the respondents stated that the majority (> 50%) of liver resections in their centre were performed laparoscopically (Fig. 1 & Supplementary Fig. 4).

In the subgroup of general surgeons, substantial experience in performing liver resections using the open approach (58.3% with > 50 resections) was threefold higher than for the laparoscopic approach (18.5% with > 50 resections). Once again, considerable experience with the robotic approach was very rare (1.8% with > 50 resections). Conversely to the findings in HPB surgeons, most general surgeons (65.5%) still preferred to use the open approach for liver resections. Furthermore, one third (34.5%) of the general surgeons stated that the open approach was the reference approach in their centre (Chosen approach in > 75% of patients) (Fig. 1 & Supplementary Fig. 4).

Intercontinental differences in the individually used approaches for colon, rectal and liver resections are portrayed in Supplementary Fig. 5.

### Usage of indocyanine green fluorescence

Overall, 38.1% of the surgeons stated that they utilized indocyanine green (ICG) fluorescence when performing colorectal resections, and 31.9% when performing liver resections. Reported ICG usage did not differ significantly between colorectal and general surgeons for colorectal resections (40.4% and 36.9%, respectively, *p* = 0.666) nor between HPB and general surgeons for liver resections (33% and 31%, respectively, *p* = 0.752). A larger proportion of the surgeons working in a non-academic hospital, however, used ICG-fluorescence when performing a colorectal resection, when compared to surgeons working in an academic hospital (47.4% vs. 30%, respectively, *p* = 0.020) (Supplementary table 1).

Furthermore, ICG fluorescence was more often adopted by respondents that currently preferred to use MIS. This correlation between respondents’ preferred approaches and ICG usage was observed for colon (open 14.3%, laparoscopic 40.8%, robotic 100%, *p* < 0.001), as well as rectal (open 17.8%, laparoscopic 38.1%, robotic 75%, Transanal TME 66.7%, *p* < 0.001) and liver surgery (Open 19%, laparoscopic 43%, hand-assisted 50%, robotic 90.9%,* p* < 0.001). Additionally, ICG was more commonly adopted by respondents from Europe and North America (Table 3).

### Surgical strategies in patients with sCRLM

In 59.3% of the respondents’ institutions, liver-first was the preferred two-stage approach in patients with an asymptomatic primary. (Table 2) Of all respondents, 72.6% had participated in a minimally invasive simultaneous resection. However, colorectal surgeons less often had first-hand experience with minimally invasive simultaneous resections when compared to HPB and general surgeons (57.9% vs. 74% and 78.8, respectively, *p* = 0.015). In most centres (57.4%), minimally invasive simultaneous resections were performed by a surgical team consisting of both a colorectal and an HPB surgeon.

When comparing the surgical strategies per continent, the liver-first two-stage approach was predominantly used in all continents except Oceania and Asia, where the colorectal primary tumour was resected first in 83.3% and 63.4% of respondents’ institutions, respectively (*p* = 0.016). MI simultaneous resections had been performed by respondents in all continents (Table 3 & supplementary Fig. 5). There were no differences in the adopted surgical strategies of surgeons that worked in an academic or non-academic setting (Supplementary table 1).

Assessing the viewpoints on simultaneous resections, many respondents would consider combining a right or left hemicolectomy with a minor (94.5% and 90.8%, respectively) or major hepatectomy (41.9% and 30.4%, respectively). (*Table **4**)* Respondents were, however, more reluctant to consider combining a low anterior or abdominoperineal resection with a minor (76.3% and 73.3%, respectively) or major hepatectomy (14.1% and 20.7%, respectively). Colorectal surgeons, compared with HPB and general surgeons, were generally more reserved in considering combining major hepatectomies with a right (22.8% vs. 50% and 44.2%, respectively, *p* = 0.008) or left hemicolectomy (14% vs. 34% and 35.4%, respectively, *p* = 0.002). A larger proportion of respondents from Oceania, Asia and North America would consider combining an abdominoperineal resection with a hepatectomy, compared with respondents from South America, Europe and Africa (83.3%, 87.5%, 76.5% vs. 66.7%, 68.2% and 62.5%, respectively, *p* = 0.029). There were no other intercontinental differences and no differences in general after stratifying for the respondents’ hospital setting (Table 5 & supplementary table 2).

**Table 4:** Viewpoints on simultaneous resection of primary colorectal cancer and CRLM overall & stratified by surgical specialty

Characteristics	Overall (*n* = 270)	CR surgery (*n* = 57)	HPB surgery (*n* = 100)	Gen. surgery (*n* = 113)	*P *value
	*n* (%)	*n* (%)	*n* (%)	*n* (%)	
*Viewpoints on simultaneous resection*					
Would consider combining:					
Right hemicolectomy with hepatectomy					0.008
No	15 (5.6)	6 (10.5)	6 (6)	3 (2.7)	
Yes, minor hepatectomy	142 (52.6)	38 (66.7)	44 (44)	60 (53.1)	
Yes, minor- and major hepatectomy	113 (41.9)	13 (22.8)	50 (50)	50 (44.2)	
Left hemicolectomy with hepatectomy					0.002
No	25 (9.3)	9 (15.8)	10 (10)	6 (5.3)	
Yes, minor hepatectomy	163 (60.4)	40 (70.2)	56 (56)	67 (59.3)	
Yes, minor and major hepatectomy	82 (30.4)	8 (14)	34 (34)	40 (35.4)	
Low anterior resection with hepatectomy					0.105
No	64 (23.7)	16 (28.1)	26 (26)	22 (19.5)	
Yes, minor hepatectomy	168 (62.2)	37 (64.9)	57 (57)	74 (65.5)	
Yes, minor and major hepatectomy	38 (14.1)	4 (7)	17 (17)	17 (15)	
Abdominoperineal resection with hepatectomy					0.077
No	72 (26.7)	17 (29.8)	30 (30)	25 (22.1)	
Yes, minor hepatectomy	142 (52.6)	34 (59.6)	46 (46)	62 (54.9)	
Yes, minor and major hepatectomy	56 (20.7)	6 (10.5)	24 (24)	26 (23)	
Would prefer creating a diverting stomy in case of MI combined resection					0.341
Never	17 (6.3)	2 (3.5)	9 (9)	6 (5.3)	
Rarely	47 (17.4)	15 (26.3)	17 (17)	15 (13.3)	
Occasionally	137 (50.7)	24 (42.1)	55 (55)	58 (51.3)	
Often	60 (22.2)	13 (22.8)	16 (16)	31 (27.4)	
Always	9 (3.3)	3 (5.3)	3 (3)	3 (2.7)	
*Opinions on outcomes after MI simultaneous versus two-stage resection*					
MI simultaneous resection carries a higher risk of postoperative complications					0.268
No, lower	30 (11.1)	2 (3.5)	13 (13)	15 (13.3)	
No, similar	127 (47)	29 (50.9)	47 (47)	51 (45.1)	
Yes	113 (41.9)	26 (45.6)	40 (40)	47 (41.6)	
MI simultaneous resection is associated with a longer length of stay					0.002
No, lower	106 (39.3)	16 (28.1)	36 (36)	54 (47.8)	
No, similar	103 (38.1)	19 (33.3)	45 (45)	39 (34.5)	
Yes	61 (22.6)	22 (38.6)	19 (19)	20 (17.7)	
MI simultaneous resection carries a higher risk of mortality					0.442
No, lower	25 (9.3)	3 (5.3)	10 (10)	12 (10.6)	
No, similar	174 (64.4)	36 (63.2)	68 (68)	70 (61.9)	
Yes	71 (26.3)	18 (31.6)	22 (22)	31 (27.4)	
Complication most worried about:					0.125
Not worried	15 (5.6)	2 (3.5)	8 (8)	5 (4.4)	
Related to the colorectal resection	122 (45.2)	18 (31.6)	53 (53)	51 (45.1)	
Related to the liver resection	38 (14.1)	8 (14)	10 (10)	20 (17.7)	
Related to both	95 (35.2)	29 (50.9)	29 (29)	37 (32.7)	
See an upcoming role for MI simultaneous resection?	250 (92.6)	51 (89.5)	96 (96)	103 (91.2)	0.241
Need for better evidence to determine feasibility and safety of MI combined approach	242 (89.6)	52 (91.2)	89 (89)	101 (89.4)	0.902

*CRLM* colorectal liver metastases, *MI* minimally invasive

**Table 5:** Viewpoints on simultaneous resection of primary colorectal cancer and CRLM per continent

Characteristics	North America (*n* = 34)	South America (*n* = 18)	Europe (*n* = 148)	Africa (*n* = 8)	Asia (*n* = 56)	Oceania (*n* = 6)	*P *value
	*n* (%)	*n* (%)	*n* (%)	*n* (%)	*n* (%)	*n* (%)	
*Viewpoints on simultaneous resection*							
Would consider combining:							
Right hemicolectomy with hepatectomy							0.750
No	/	2 (11.1)	11 (7.4)	1 (12.5)	/	1 (16.7)	
Yes, minor hepatectomy	12 (35.3)	9 (50)	88 (59.5)	3 (37.5)	27 (48.2)	3 (50)	
Yes, minor and major hepatectomy	22 (64.7)	7 (38.9)	49 (33.1)	4 (50)	29 (51.8)	2 (33.3)	
Left hemicolectomy with hepatectomy							0.331
No	/	3 (16.7)	15 (10.1)	4 (50)	2 (3.6)	1 (16.7)	
Yes, minor hepatectomy	16 (47.1)	13 (72.2)	94 (63.5)	3 (37.5)	32 (57.1)	5 (83.3)	
Yes, minor and major hepatectomy	18 (52.9)	2 (11.1)	39 (26.4)	1 (12.5)	22 (39.3)	/	
Low anterior resection with hepatectomy							0.579
No	3 (8.8)	6 (33.3)	41 (27.7)	4 (50)	9 (16.1)	1 (16.7)	
Yes, minor hepatectomy	21 (61.8)	10 (55.6)	94 (63.5)	1 (12.5)	37 (66.1)	5 (83.3)	
Yes, minor and major hepatectomy	10 (29.4)	2 (11.1)	13 (8.8)	3 (37.5)	10 (17.9)	/	
Abdominoperineal resection with hepatectomy							0.029
No	8 (23.5)	6 (33.3)	47 (31.8)	3 (37.5)	7 (12.5)	1 (16.7)	
Yes, minor hepatectomy	18 (52.9)	12 (66.7)	74 (50)	3 (37.5)	32 (57.1)	3 (50)	
Yes, minor and major hepatectomy	8 (23.5)	/	27 (18.2)	2 (25)	17 (30.4)	2 (33.3)	
Would prefer creating a diverting stomy in case of MI combined resection							0.053
Never	5 (14.7)	2 (11.1)	7 (4.7)	1 (12.5)	2 (3.6)	/	
Rarely	10 (29.4)	1 (5.6)	24 (16.2)	1 (12.5)	10 (17.9)	1 (16.7)	
Occasionally	12 (35.3)	10 (55.6)	82 (55.4)	3 (37.5)	27 (48.2)	3 (50)	
Often	6 (17.6)	3 (16.7)	31 (20.9)	3 (37.5)	15 (26.8)	2 (33.3)	
Always	1 (2.9)	2 (11.1)	4 (2.7)	/	2 (3.6)	/	
*Opinions on outcomes after MI simultaneous versus two-staged resection*							
MI simultaneous resection carries a higher risk of postoperative complications							0.883
No, lower	4 (11.8)	2 (11.1)	16 (10.8)	1 (12.5)	7 (12.5)	/	
No, similar	18 (52.9)	10 (55.6)	62 (41.9)	6 (75)	29 (51.8)	2 (33.3)	
Yes	12 (35.3)	6 (33.3)	70 (47.3)	1 (12.5)	20 (35.7)	4 (66.7)	
MI simultaneous resection is associated with a longer length of stay							0.917
No, lower	10 (29.4)	6 (33.3)	65 (43.9)	6 (75)	18 (32.1)	1 (16.7)	
No, similar	16 (47.1)	8 (44.4)	50 (33.8)	1 (12.5)	24 (42.9)	4 (66.7)	
Yes	8 (23.5)	4 (22.2)	33 (22.3)	1 (12.5)	14 (25)	1 (16.7)	
MI simultaneous resection carries a higher risk of mortality							0.727
No, lower	4 (11.8)	2 (11.1)	13 (8.8)	/	5 (8.9)	1 (16.7)	
No, similar	22 (64.7)	12 (66.7)	95 (64.2)	5 (62.5)	36 (64.3)	4 (66.7)	
Yes	8 (23.5)	4 (22.2)	40 (27)	3 (37.5)	15 (26.8)	1 (16.7)	
Complication most worried about:							0.174
Not worried	3 (8.8)	1 (5.6)	6 (4.1)	/	5 (8.9)	/	
Related to the colorectal resection	15 (44.1)	10 (55.6)	70 (47.3)	5 (62.5)	19 (33.9)	3 (50)	
Related to the liver resection	7 (20.6)	2 (11.1)	20 (13.5)	/	9 (16.1)	/	
Related to both	9 (26.5)	5 (27.8)	52 (35.1)	3 (37.5)	23 (41.1)	3 (50)	
See an upcoming role for MI simultaneous resection?	33 (97.1)	17 (94.4)	140 (94.6)	5 (62.5)	51 (91.1)	4 (66.7)	0.002
Need for better evidence to determine feasibility and safety of MI combined approach	29 (85.3)	16 (88.9)	133 (89.9)	7 (87.5)	51 (91.1)	6 (100)	0.905

*CRLM* colorectal liver metastases, *MDT* multidisciplinary team, *MI* minimally invasive

The respondents expressed several concerns regarding minimally invasive simultaneous resections, although significant heterogeneity was present. While many respondents believed that the risk of morbidity and mortality was comparable with the two-stage approach (47% and 64.4%, respectively), others associated minimally invasive simultaneous resections with a higher risk of postoperative morbidity (41.9%) and mortality (26.3%). Furthermore, 38.1% of the respondents associated this procedure with a shorter LOS, while, conversely, 22.6% of the respondents believed that minimally invasive simultaneous resections were associated with a longer LOS. Colorectal surgeons more often stated that they thought that minimally invasive simultaneous resections were associated with a longer length of stay than HPB specialists and general surgeons (38.6% vs. 19% and 17.7%, respectively, *p* = 0.002).

Further addressing specific concerns related to minimally invasive simultaneous resections, 45.2% of the respondents was concerned about postoperative morbidity related to the colorectal resection, while the other respondents were worried about morbidity related to the liver resection (14.1%), both procedures (35.2%) or not worried at all (5.6%). Despite these worries, an upcoming role for minimally invasive simultaneous resections in the surgical management of this patient population was foreseen by 92.6% of the respondents, albeit that surgeons from Africa and Oceania less often foresaw an expanding role for this procedure (62.5% and 66.7% vs. 91.1–97.1%, respectively, *p* = 0.002). (*Table **5*) Additionally, further evidence to determine the feasibility and safety of this procedure was deemed necessary (89.6%). (*Table **4*).

### Developments in the treatment of CRC and CRLM

When asked which developments in the management of CRC have made the most clinical impact over the past two decades, “the movement towards and pushing the boundaries in MIS” was selected by most respondents (33%), followed by “improvement of systemic treatments and their usage” (27.4%), “better understanding of molecular biology and thereby possibility to provide personalized medicine” (24.8%), “usage of enhanced recovery after surgery (ERAS) protocols” (10.7%), and “improved imaging modalities” (3%) (Supplementary Fig. 6).

Essential developments in the management of CRLM were deemed to include “the Introduction of aggressive surgical approaches” (26.3%) and “improvement of systemic treatments and their usage” (25.6%), followed by “movement towards parenchymal-sparing resections” (21.9%), “better understanding of molecular biology and thereby possibility to provide personalized medicine” (13.7%), “movement towards and pushing the boundaries of MIS” (7.4%), “usage of ERAS protocols” (2.6%), and “usage of thermal ablation” (1.5%).

## Discussion

This international survey study aimed to assess the current practices and viewpoints of surgeons involved in the treatment of patients with sCRLM. Although there is a general shift towards specialization within the surgical community, this study shows that the surgical care for this patient population is still provided by a mixture of general and specialist surgeons depending on local and institutional standards [16]. The study also confirms that there are differences among these groups of health care providers in terms of attitudes and preferences, while consensus on the optimal treatment strategy for patients with sCRLM is lacking. At the same time, there appears to be consensus on the growing role for MIS and a need for evidence-based input.

Despite the substantial amount of evidence confirming the feasibility, safety and oncological efficiency of the laparoscopic approach for both colorectal and liver disease, we herein note that MIS is not yet the gold standard in both fields [11–13, 17–25]. While the majority of the respondents preferred to use the laparoscopic approach for colon resections, this technique was less often selected for rectal or liver resections. The variation in these preference rates is possibly caused by the fact that rectal and liver resections are technically more demanding, with a longer learning curve [26, 27]. For rectal resections, another contributing factor may be the heterogeneity of the published data of randomized controlled trials on the oncological safety of MIS [28–32]. Specifically looking at liver surgery, the adoption of MIS has been rather slow because of worries about haemorrhage control and its oncological safety. [19]

Concerning practice variation, general surgeons reported a less frequent use of MIS and, despite not statistically significant, ICG usage during colorectal and liver resections. This suggests that specialists have a higher tendency to introduce innovative approaches and techniques while general surgeons seem less inclined to adopt innovative practices [33]. Additionally, general surgeons who utilized MIS often had more limited experience with these approaches when compared to specialists. This finding is possibly because specialization is more likely to be implemented in high volume centres, hence, leading to more surgical experience with a single organ. These findings are of particular interest, since it has been known for many years that both specialization and a higher surgeon volume may be associated with improved clinical and possibly even oncological outcomes [34–36]. Nevertheless, our study shows that general surgeons still play an important role in the management of these patients.

When managing patients with sCRLM, the majority of both general and specialist surgeons seem to function within a “treatment team,” in agreement with strong recommendations in guidelines and evidence associating multidisciplinary treatment planning with superior outcomes [14, 15, 37]. However, not all respondents had access to an MDT where patients affected by sCRLM were discussed, suggesting that this is not yet considered mandatory. Additionally, there were significant differences in the type of MDT where patients with sCRLM were discussed. While a large proportion of the respondents would collaborate when planning the surgical management strategy for patients with sCRLM eligible for resection of both the primary CRC and CRLM, general surgeons and surgeons working in a non-academic setting were more inclined to take sole decisions in this regard. Lastly, and of concern, almost 1 in 10 respondents described the working relationship with their colleagues from another specialty as suboptimal. Thus, while the results of this survey indicate that in many centres integrated care pathways have been established for patients with sCRLM, there is room for improvement. Standardizing the composition and workflow of MDTs and expanding and enhancing interdisciplinary collaboration, especially between general and specialist surgeons, seems desirable.

Regarding the used surgical timing strategies in patients with synchronous disease, the results of this survey reflect the currently available evidence, which so far has not shown that one strategy is adopted more and or considered “superior” to others. All three available strategies (colorectal-first, liver-first and simultaneous resection) seem to be utilized on a global scale, and by surgeons working in both academic and non-academic hospitals [7, 9]. Liver-first was the preferred two-stage approach in a slight majority of the respondents’ institutions. Regional differences were, however, observed; in centres in Asia and Oceania, the colorectal-first strategy was preferred, while this was the liver-first approach in the rest of the world. As reported before, the liver-first approach, therefore, now seems widely dispersed, even though its hypothesized oncological superiority has never been confirmed in the overall population of patients with resectable sCRLM [38]. Nevertheless, recent research seems to indicate that specific surgical strategies should be decided based on a patients’ hepatic disease burden, as a large registry-based study showed that patients with multiple bilobar metastases gained a survival benefit from a liver-first approach [38]. Individualized treatment plans, preferably established by a multidisciplinary team, therefore, remain key in the management of these patients [14, 15].

Assessing the viewpoints of respondents on simultaneous resections, respondents were especially reluctant to consider combining low anterior or abdominoperineal resections with a major hepatectomy. Taking into account that the risk for postoperative morbidity and mortality after a simultaneous resection seems to increase with the complexity of the colorectal procedure and the extent of the liver resection, this seems a rational standpoint [39, 40]. Rectal resections can be lengthy and complex in addition to the added risk by, if utilized, neoadjuvant chemoradiotherapy [41–44]. However, low anterior resections were identified earlier as “low risk” colorectal procedures. Therefore, the overall reluctancy to combine this procedure with a hepatectomy needs some further reflections [39].

For this reluctancy, several possible reasons were noted, as a substantial proportion of the respondents believed minimally invasive simultaneous resections could lead to a higher risk of postoperative mortality, morbidity and a longer LOS. Contradictory to these beliefs, a plethora of studies comparing the outcomes following two-staged and simultaneous resections have reported similar postoperative mortality rates and a shorter LOS after simultaneous resections [7, 40]. The current evidence is, however, less clear on the risk of postoperative morbidity following simultaneous resections, since some studies have associated the simultaneous approach with a higher risk of morbidity while others have found this risk to be comparable to the two-staged approach [7, 9, 39, 40]. There was little consensus present between the different groups of healthcare providers, as colorectal specialists were less inclined to combine hemicolectomies with major hepatectomies and in general had a more negative view on the simultaneous approach.

Following the earlier mentioned growing body of evidence of the merits of minimally invasive liver surgery in patients with CRLM and the routine usage of MIS for CRC resections, a larger role for minimally invasive simultaneous resections in the management of this patient population seems logical. In our study, this was also the general opinion, albeit to a lesser extent in Africa and Oceania, as 92.6% of all respondents saw an expanding role for this procedure in the future. A recent meta-analysis supports this ambition, reporting several advantages of minimally invasive, compared to open, simultaneous resections: a lower postoperative morbidity rate, less intraoperative blood loss and a shorter length of hospital stay [10]. Currently the role of this procedure, however, seems limited, probably due to the scarce amount of data on the subject as stated in the Southampton consensus guidelines in 2017 and in a recently published Italian National Consensus statement [40, 45, 46]. A need for additional evidence in this field was also expressed by a large proportion of the respondents in this study.

This study has various limitations. First, the number of respondents, especially specialized colorectal surgeons, was limited. This could be related to the fact that only one multinational society of colorectal surgeons decided to support the project. In addition, colorectal surgeons working in centres without a liver surgery unit were less likely to respond because of the content and subject of the survey. Second, the total number of surgeons who have received an invitation to complete the survey, and therefore, the response rate, is unknown. The memberships of the involved societies are partially overlapping, and membership lists, including the number of specialized surgeons per society, cannot be reviewed. Third, a considerable proportion of the respondents was from Europe and the United States, and therefore, the results may not completely reflect a global experience. Fourth, this study does not delve into details regarding, for example, peri-operative management since the aim of this study was, as a first step, to gain an overview of the context in which surgeons manage these patients (in terms of team composition, utilized approaches, surgical timing etc.).

## Conclusion

The surgical management of patients with sCRLM is rapidly evolving. Although health care providers often work in a multidisciplinary setting, their viewpoints differ in some respects between continents, and between and within surgical specialties. Nevertheless, there appears to be consensus on an expanding role for MIS and simultaneous resections. Additional evidence in this field, preferably in the form of multicentre randomized controlled trials and (inter)national registries, could bring stakeholders even closer together and improve the overall quality of surgical care for this patient population.

## Supplementary Information

Below is the link to the electronic supplementary material.

Supplementary file1 (DOCX 30 kb)

Supplementary file2 (DOCX 36 kb)

Supplementary file3 (TIFF 4806 kb)—Supplementary figure 1. Number of respondents per country

Supplementary file4 (TIFF 33592 kb)—Supplementary figure 2. Volume and institutional usage of the different surgical approaches for colon resections. A) Personal volume of colorectal surgeons B) Utilized surgical approaches in colorectal surgeons’ institutions C) Personal volume of general surgeons D) Utilized surgical approaches in general surgeons’ institutions

Supplementary file5 (TIFF 33566 kb)—Supplementary figure 3. Volume and institutional usage of the different surgical approaches for rectal resections. A) Personal volume of colorectal surgeons B) Utilized approaches in colorectal surgeons’ institutions C) Personal volume of general surgeons D) Utilized approaches in general surgeons’ institutions

Supplementary file6 (TIFF 33592 kb)—Supplementary figure 4. Volume and institutional usage of the different surgical approaches for liver resections. A) Personal volume of HPB surgeons B) Utilized surgical approaches in HPB surgeons’ institutions C) Personal volume of general surgeons D) Utilized surgical approaches in general surgeons’ institutions

Supplementary file7 (TIFF 33592 kb)—Supplementary figure 5. Countries of residence of respondents and intercontinental differences in used approaches and strategies in patients with sCRLM

Supplementary file6 (TIFF 33592 kb)—Supplementary figure 6. Viewpoints of respondents on the developments in the treatment of colorectal cancer and colorectal liver metastases which made the most clinical impact over the last two decades
