# Supplementary material for: Global survey on the surgical management of patients affected by colorectal cancer with synchronous liver metastases: impact of surgical specialty and geographic region
Source: Surg Endosc. 2023 Mar 6;37(6):4658–72. doi: 10.1007/s00464-023-09917-8 (PMC10234876; doi:10.1007/s00464-023-09917-8)
Supplement: Supplementary file 1 — Supplementary file1 (DOCX 30 kb) [file 464_2023_9917_MOESM1_ESM.docx]

| **Supplementary table 1.** Organization of care, opinions on clinical judgement and surgical strategies for patients with CRLMs stratified by hospital setting (academic versus non-academic) of respondent | | | |  |
| --- | --- | --- | --- | --- |
| *Characteristics* | Academic (n=179) | Non-academic (n=91) | P-value |  |
|  | N (%) / Median (IQR) | N (%) / Median (IQR) |  |  |
| *Organization of care for patients with CRLMs* |  |  |  |  |
| Access to MDT-meeting | 169 (94.4) | 87 (95.6) | 0.677 |  |
| Type of MDT |  |  | 0.003 |  |
| Colorectal | 14 (8.3) | 5 (5.7) |  |  |
| HPB | 26 (15.4) | 8 (9.2) |  |  |
| Shared | 56 (33.1) | 50 (57.5) |  |  |
| Both | 73 (43.2) | 24 (27.6) |  |  |
| Discuss cases with respective colleague before the MDT (**n=188**) * |  |  | 0.707 |  |
| <50% of cases | 41 (33.1) | 22 (34.4) |  |  |
| 50-99% of cases | 26 (21) | 15 (23.4) |  |  |
| 100% of cases | 57 (46) | 27 (42.2) |  |  |
| After MDT plan overall surgical management with respective colleague(s) (**n=256**) | 151 (89.3) | 69 (79.3) | 0.029 |  |
| No access, but will discuss cases with respective colleague(s) (**n=14**) |  |  | 0.071 |  |
| <50% of cases | 4 (40) | 4 (100) |  |  |
| 50-99% of cases | 3 (30) | / |  |  |
| 100% of cases | 3 (30) | / |  |  |
| *Working relationship with respective colleagues* |  |  | 0.037 |  |
| Excellent | 120 (67) | 51 (56) |  |  |
| Good | 41 (22.9) | 24 (26.4) |  |  |
| Adequate | 11 (6.1) | 8 (8.8) |  |  |
| Fair | 2 (1.1) | 2 (2.2) |  |  |
| Poor | 1 (0.6) | 2 (2.2) |  |  |
| No respective colleague in institution | 4 (2.2) | 4 (4.4) |  |  |
| *Opinions on clinical judgement* |  |  |  |  |
| Able to determine eligibility for local treatment? |  |  | 0.491 |  |
| No | 6 (3.4) | 2 (2.2) |  |  |
| Solely | 33 (18.4) | 16 (17.6) |  |  |
| In context of MDT | 114 (63.7) | 65 (71.4) |  |  |
| Together with a radiologist | 26 (14.5) | 8 (8.8) |  |  |
| HPB surgeons best suited to determine eligilbity for local treatment? |  |  | 0.614 |  |
| Yes | 166 (92.7) | 87 (95.6) |  |  |
| Only in context of MDT | 5 (2.8) | 2 (2.2) |  |  |
| Others are best suited | 8 (4.5) | 2 (2.2) |  |  |
| *Surgical strategies* |  |  |  |  |
| Two-staged procedure in patient with asymptomatic primary |  |  | 0.808 |  |
| Colorectal first | 72 (40.2) | 38 (41.8) |  |  |
| Liver-first | 107 (59.8) | 53 (58.2) |  |  |
| Usage of ICG-fluorescence |  |  |  |  |
| Colorectal resections (**n=168**) | 27 (30) | 37 (47.4) | 0.020 |  |
| Liver resections (**n=213**) | 46 (29.9) | 22 (37.3) | 0.299 |  |
| Participated in a MI simultaneous resection | 131 (73.2) | 65 (71.4) | 0.760 |  |
| *Strategies for simultaneous resection* |  |  |  |  |
| In my institution, MI simultaneous resection is performed by: |  |  | 0.160 |  |
| Not performed yet | 35 (19.6) | 18 (19.8) |  |  |
| Colorectal surgeon & HPB-surgeon together | 110 (61.5) | 45 (49.5) |  |  |
| HPB-surgeon with experience in colorectal resections | 19 (10.6) | 16 (17.6) |  |  |
| Colorectal surgeon with experience in liver resections | 15 (8.4) | 12 (13.2) |  |  |
| Number of surgeons performing MI combined resection in institution |  |  | 0.320 |  |
| Zero | 20 (29.9) | 6 (13) |  |  |
| One | 11 (16.4) | 9 (19.6) |  |  |
| Two | 17 (25.4) | 19 (41.3) |  |  |
| Three | 14 (20.9) | 11 (23.9) |  |  |
| Four or more | 5 (7.5) | 1 (2.2) |  |  |
| Abbreviations: CRLM: colorectal liver metastases, MDT; multidisciplinary team, MI; minimally invasive  *For colorectal specialists this question was only asked when the “colorectal” or “both” type of MDT was chosen, for HPB- subspecialists when the “HPB” or “both” option was chosen. | | | |  |
|  |  |  |  |  |

| **Supplementary table 2.** Viewpoints on simultaneous resection of primary colorectal cancer and CRLMs stratified by hospital setting (academic versus non-academic) of respondent | | | |  |
| --- | --- | --- | --- | --- |
| *Characteristics* | Academic | Non-academic | P-value |  |
|  | N (%) / Median (IQR) | N (%) / Median (IQR) |  |  |
| *Viewpoints on simultaneous resection* |  |  |  |  |
| Would consider combining: |  |  |  |  |
| Right hemicolectomy with hepatectomy |  |  | 0.656 |  |
| No | 10 (5.6) | 5 (5.5) |  |  |
| Yes, minor hepatectomy | 92 (51.4) | 50 (54.9) |  |  |
| Yes, minor- and major hepatectomy | 77 (43) | 36 (39.6) |  |  |
| Left hemicolectomy with hepatectomy |  |  | 0.964 |  |
| No | 15 (8.4) | 10 (11) |  |  |
| Yes, minor hepatectomy | 111 (62) | 52 (57.1) |  |  |
| Yes, minor- and major hepatectomy | 53 (29.6) | 29 (31.9) |  |  |
| Low anterior resection with hepatectomy |  |  | 0.370 |  |
| No | 38 (21.2) | 26 (28.6) |  |  |
| Yes, minor hepatectomy | 116 (64.8) | 52 (57.1) |  |  |
| Yes, minor- and major hepatectomy | 25 (14) | 13 (14.3) |  |  |
| Abdominoperineal resection with hepatectomy |  |  | 0.941 |  |
| No | 46 (25.7) | 26 (28.6) |  |  |
| Yes, minor hepatectomy | 98 (54.7) | 44 (48.4) |  |  |
| Yes, minor- and major hepatectomy | 35 (19.6) | 21 (23.1) |  |  |
| Would prefer creating a diverting stomy in case of MI combined resection |  |  | 0.999 |  |
| Never | 13 (7.3) | 4 (4.4) |  |  |
| Rarely | 29 (16.2) | 18 (19.8) |  |  |
| Occasionally | 90 (50.3) | 47 (51.6) |  |  |
| Often | 41 (22.9) | 19 (20.9) |  |  |
| Always | 6 (3.4) | 3 (3.3) |  |  |
| *Opinions on outcomes after MI simultaneous versus two-staged resection* |  |  |  |  |
| MI simultaneous resection carries a higher risk of postoperative complications |  |  | 0.996 |  |
| No, lower | 18 (10.1) | 12 (13.2) |  |  |
| No, similar | 88 (49.2) | 39 (42.9) |  |  |
| Yes | 73 (40.8) | 40 (44.0) |  |  |
| MI simultaneous resection is associated with a longer length of stay |  |  | 0.759 |  |
| No, lower | 68 (38) | 38 (41.8) |  |  |
| No, similar | 71 (39.7) | 32 (35.2) |  |  |
| Yes | 40 (22.3) | 21 (23.1) |  |  |
| MI simultaneous resection carries a higher risk of mortality |  |  | 0.432 |  |
| No, lower | 15 (8.4) | 10 (11) |  |  |
| No, similar | 122 (68.2) | 52 (57.1) |  |  |
| Yes | 42 (23.5) | 29 (31.9) |  |  |
| Complication most worried about: |  |  | 0.679 |  |
| Not worried | 12 (6.7) | 3 (3.3) |  |  |
| Related to the colorectal resection | 79 (44.1) | 43 (47.3) |  |  |
| Related to the liver resection | 25 (14.5) | 12 (13.2) |  |  |
| Related to both | 62 (34.6) | 33 (36.3) |  |  |
| See an upcoming role for MI simultaneous resection? | 166 (92.7) | 84 (92.3) | 0.899 |  |
| Need for better evidence to determine feasibility and safety of MI combined approach | 162 (90.5) | 80 (87.9) |  |  |
| Abbreviations: CRLM: colorectal liver metastases, MDT; multidisciplinary team, MI; minimally invasive | | | |  |
|  |  |  |  |  |
