# Supplementary material for: Global survey on the surgical management of patients affected by colorectal cancer with synchronous liver metastases: impact of surgical specialty and geographic region
Source: Surg Endosc. 2023 Mar 6;37(6):4658–72. doi: 10.1007/s00464-023-09917-8 (PMC10234876; doi:10.1007/s00464-023-09917-8)
Supplement: Supplementary file 2 — Supplementary file2 (DOCX 36 kb) [file 464_2023_9917_MOESM2_ESM.docx]

**Global survey on current practices in the surgical management of colorectal cancer with synchronous liver metastases**

**Basic information**

Country : ……………………………………

Institution : ……………………………………

Years of experience as a surgeon : ……………………………………….

Name : ……………………………………

E-mail : ……………………………………

**Introductory questions**

1. In your opinion, what development in the management of colorectal cancer over the past two decades has made the most clinical impact?
   1. Usage of enhanced recovery after surgery (ERAS) protocols
   2. Movement towards and pushing the boundaries of minimally invasive surgery
   3. Improvement of systemic treatment and their usage (e.g., chemotherapeutic regimens)
   4. Better understanding of molecular biology and thereby possibility to provide personalized medicine
   5. Improved imaging modalities
   6. Other: ……….
2. In your opinion, what development in the management of colorectal liver metastases over the past two decades has made the most clinical impact?
   1. Usage of enhanced recovery after surgery (ERAS) protocols
   2. Movement towards and pushing the boundaries of minimally invasive surgery
   3. Improvement of systemic treatment and their usage (e.g., chemotherapeutic regimens)
   4. Better understanding of molecular biology and thereby possibility to provide personalized medicine
   5. Movement towards parenchymal-sparing resection
   6. Introduction of aggressive surgical approaches (e.g., multiple stage hepatectomies, ALPPS)
   7. Usage of thermal ablation
   8. Other: ……….
3. In what type of hospital do you work?
   1. Academic
   2. Non-Academic teaching
   3. Non-teaching
   4. Other: ……….
4. Regarding the scope of this survey: what type of surgery do you perform?
   1. Only colorectal surgery 🡪 if specified, only questions labelled (C) were presented
   2. Only HPB surgery 🡪 if specified, only questions labelled (H) were presented
   3. Both colorectal and liver surgery 🡪 if specified, only questions labelled (G) were presented
5. How many colorectal cancer resections are performed yearly in your institution? (C,G)

±……………….

1. How many colorectal cancer resections do you perform per year? (C,G)

±……………….

1. How many liver resections are performed yearly in your institution? (H,G)

±……………….

1. How many liver resections do you perform per year? (H,G)

±……………….

**Working relationship between HPB and colorectal surgeons – section for general surgeons**

1. Do you have access to a multidisciplinary team (MDT) meeting where patients with CRLMs are discussed?
   1. Yes, go to question 11
   2. No, go to question 10
2. In which percentage of cases with known synchronous CRLMs do you discuss the overall surgical management plan with another HPB or colorectal surgeon?
   1. 100%
   2. 50-99%
   3. <50%

🡪 go to question 14

1. In which type of MDT are patients with CRLMs discussed?
   1. Colorectal MDT meeting
   2. HPB MDT meeting
   3. Shared MDT meeting
   4. In both a CRC and HPB MDT meeting
2. What percentage of new patients with synchronous CRLMs do you decide to discuss with another HPB or colorectal surgeon prior to the MDT meeting?
   1. 100%
   2. 50-99%
   3. <50%
3. During the MDT meeting a patient with synchronous CRLMs with the primary colorectal tumour still in situ is discussed. Resection of both the primary tumour and CRLMs is advised. Do you plan the surgical management strategy together with another colorectal or HPB surgeon?
   1. Yes
   2. No
4. How do you consider the working relationship with the other HPB- and colorectal surgeons in your institution?
   1. Excellent, go to question 30
   2. Good, go to question 30
   3. Adequate, go to question 30
   4. Fair, go to question 15
   5. Poor, go to question 15
5. Why do you consider this working relationship suboptimal?
   1. We work independently of each other
   2. Different views on treatment strategies
   3. Poor communication
   4. Other: ……….

🡪Go to question 30

**Working relationship between HPB and colorectal surgeons – section for colorectal surgeons**

1. Do you have access to a multidisciplinary team (MDT) meeting where patients with CRLMs are discussed?
   1. Yes, go to question 17
   2. No, go to question 18
2. In which percentage of cases with known synchronous CRLMs do you discuss the overall surgical management plan with another HPB or colorectal surgeon?
   1. 100%
   2. 50-99%
   3. <50%

🡪 go to question 21

1. In which type of MDT are patients with CRLMs discussed?
   1. Colorectal MDT meeting 🡪 go to question 19
   2. HPB MDT meeting 🡪 go to question 20
   3. Shared MDT meeting 🡪 go to question 20
   4. In both a CRC and HPB MDT meeting 🡪 go to question 19
2. What percentage of new patients with synchronous CRLMs do you decide to discuss with an HPB surgeon prior to the MDT meeting?
   1. 100%
   2. 50-99%
   3. <50%
3. During the MDT meeting a patient with synchronous CRLMs with the primary colorectal tumour still in situ is discussed. Resection of both the primary tumour and CRLMs is advised. Do you plan the surgical management strategy together with an HPB surgeon?
   1. Yes
   2. No
4. How do you consider the working relationship with the HPB surgeons in your institution?
   1. Excellent, go to question 30
   2. Good, go to question 30
   3. Adequate, go to question 30
   4. Fair, go to question 22
   5. Poor, go to question 22
5. Why do you consider this working relationship suboptimal?
   1. We work independently of each other
   2. Different views on treatment strategies
   3. Poor communication
   4. Other: ……….

🡪 go to question 30

**Working relationship between HPB and colorectal surgeons – section for HPB surgeons**

1. Do you have access to a multidisciplinary team (MDT) meeting where patients with CRLMs are discussed?
   1. Yes, go to question 25
   2. No, go to question 24
2. In which percentage of cases with known synchronous CRLMs do you discuss the overall surgical management plan with a colorectal surgeon?
   1. 100%
   2. 50-99%
   3. <50%

🡪 go to question 28

1. In which type of MDT are patients with CRLMs discussed?
   1. Colorectal MDT meeting 🡪 go to question 27
   2. HPB MDT meeting 🡪 go to question 26
   3. Shared MDT meeting 🡪 go to question 27
   4. In both a CRC and HPB MDT meeting 🡪 go to question 26
2. What percentage of new patients with synchronous CRLMs do you decide to discuss with an HPB surgeon prior to the MDT meeting?
   1. 100%
   2. 50-99%
   3. <50%
3. During the MDT meeting a patient with synchronous CRLMs with the primary colorectal tumour still in situ is discussed. Resection of both the primary tumour and CRLMs is advised. Do you plan the surgical management strategy together with an HPB surgeon?
   1. Yes
   2. No
4. How do you consider the working relationship with the colorectal surgeons in your institution?
   1. Excellent, go to question 30
   2. Good, go to question 30
   3. Adequate, go to question 30
   4. Fair, go to question 29
   5. Poor, go to question 29
5. Why do you consider this working relationship suboptimal?
   1. We work independently of each other
   2. Different views on treatment strategies
   3. Poor communication
   4. Other: ……….

**Surgical management**

1. How many liver resections have you performed in total? (H,G)

|  | 0-25 | 25-50 | 50-75 | 75-100 | 100+ |
| --- | --- | --- | --- | --- | --- |
| Open |  |  |  |  |  |
| Laparoscopic |  |  |  |  |  |
| Robotic |  |  |  |  |  |

1. In the past 3 years combined what percentage of liver resections is performed using which technique in your centre? (H,G)

|  | 0-25% | 25-50% | 50-75% | 75-100% |
| --- | --- | --- | --- | --- |
| Open |  |  |  |  |
| Laparoscopic |  |  |  |  |
| Robotic |  |  |  |  |

1. What surgical technique do you currently usually use when performing liver resections? (H,G)
   1. Open
   2. Laparoscopic
   3. Robotic
   4. Hand assisted
2. Do you use ICG fluorescence when performing liver resections and if so for what reason? (H,G) 🡪 can choose multiple options
   1. Yes, for anatomical demarcation
   2. Yes, to define tumour borders
   3. Yes, to identify occult metastases
   4. No, I do not use ICG fluorescence during liver resections
3. How many colon resections have you performed in total? (C,G)

|  | 0-25 | 25-50 | 50-75 | 75-100 | 100+ |
| --- | --- | --- | --- | --- | --- |
| Open |  |  |  |  |  |
| Laparoscopic |  |  |  |  |  |
| Robotic |  |  |  |  |  |

1. In the past 3 years combined what percentage of colon resections was performed using which technique in your centre? (C,G)

|  | 0-25% | 25-50% | 50-75% | 75-100% |
| --- | --- | --- | --- | --- |
| Open |  |  |  |  |
| Laparoscopic |  |  |  |  |
| Robotic |  |  |  |  |

1. What surgical technique do you currently usually use when performing resection of a colon tumour? (C,G)
   1. Open
   2. Laparoscopic
   3. Robotic
   4. Hand assisted
2. How many rectal resections have you performed in total? (C,G)

|  | 0-25 | 25-50 | 50-75 | 75-100 | 100+ |
| --- | --- | --- | --- | --- | --- |
| Open |  |  |  |  |  |
| Laparoscopic |  |  |  |  |  |
| Robotic |  |  |  |  |  |
| Transanal TME |  |  |  |  |  |

1. In the past 3 years combined what percentage of rectal resections was performed using which technique in your centre? (C,G)

|  | 0-25% | 25-50% | 50-75% | 75-100% |
| --- | --- | --- | --- | --- |
| Open |  |  |  |  |
| Laparoscopic |  |  |  |  |
| Robotic |  |  |  |  |
| Transanal TME |  |  |  |  |

1. What surgical technique do you currently usually use when performing resection of a rectal tumour? (C,G)
   1. Open
   2. Laparoscopic
   3. Robotic
   4. Hand assisted
   5. Transanal TME
2. Do you use ICG fluorescence when performing colorectal resections and if so for what reason? (C,G)
   1. Yes, for guidance when performing lymphadenectomies
   2. Yes, to assess vascularization
   3. Yes, for both options above
   4. No, I do not use ICG fluorescence during colorectal resections

1. Do you believe that you are able to determine if CRLMs are eligible for local treatment?
   1. Yes, solely
   2. Yes, in collaboration with a radiologist
   3. Yes, in the context of the MDT
   4. No
2. Do you believe that HPB surgeons are best suited to determine if CRLMs are eligible for local treatment?
   1. Yes
   2. No, …………………………………………………………… are best suited
3. When performing a two-staged resection in patients with synchronous CRLMs and an asymptomatic primary tumour, what is the routine surgical approach in your institution?
   1. Liver first
   2. Colorectal first
4. In how many open combined resections (simultaneous resection of primary CRC and CRLMs) have you participated/do you participate in patients with synchronous CRLMs in total/annually?

Total : ……………………………………………

Annually : ……………………………………………

1. Do you see an upcoming role for minimally invasive (MI) combined resection in the management of synchronous CRLMs?
   1. Yes
   2. No
2. Have you ever participated in a MI combined resection?
   1. Yes
   2. No, go to question 48
3. In how many MI combined resections have you participated/do you participate in total/annually?

Total : ………………………………………………..

Annually : ………………………………………………..

1. How many surgeons perform MI combined resection in your institution?
   1. 0
   2. 1
   3. 2
   4. 3
   5. ≥ 4
2. By whom is the MI combined resection performed in your institution?
   1. Colorectal surgeon with experience in liver resections
   2. HPB surgeon and colorectal surgeon together
   3. HPB surgeon with experience in colorectal resections
   4. It has not (yet) been performed in my institution
3. Would you consider performing a minor (≤2 segments) or major (≥3 segments) hepatic resection combined with a right hemicolectomy?
   1. No
   2. Yes, minor hepatectomy only
   3. Yes, minor and major hepatectomy
4. Would you consider performing a minor (≤2 segments) or major (≥3 segments) hepatic resection combined with a left hemicolectomy?
   1. No
   2. Yes, minor hepatectomy only
   3. Yes, minor and major hepatectomy
5. Would you consider performing a minor (≤2 segments) or major (≥3 segments) hepatic resection combined with a low anterior resection?
   1. No
   2. Yes, minor hepatectomy only
   3. Yes, minor and major hepatectomy
6. Would you consider performing a minor (≤2 segments) or major (≥3 segments) hepatic resection combined with an abdominoperineal resection?
   1. No
   2. Yes, minor hepatectomy only
   3. Yes, minor and major hepatectomy
7. In case of a MI combined resection, I would prefer it if a (temporary) diverting ileo/colostomy is created:
   1. Never
   2. Occasionally
   3. Rarely
   4. Often
   5. Always

**Opinion on outcomes**

1. Do you believe that a MI combined resection carries a higher risk of postoperative complications?
   1. Yes
   2. No, similar risk of postoperative complications
   3. No, lower risk of postoperative complications
2. Do you believe that a MI combined resection is associated with a longer length of hospital stay compared to the two-staged approach(cumulative)?
   1. Yes
   2. No, similar length of hospital stay
   3. No, shorter length of hospital stay
3. Which postoperative complication would you be most worried of after a MI combined resection?
   1. Liver related
   2. Colorectal related
   3. Colorectal and liver related
   4. I am not worried of postoperative complications
4. Do you believe that MI combined resection carries a higher risk of mortality compared to the two-staged approach?
   1. Yes
   2. No, similar risk of mortality
   3. No, lower risk of mortality
5. Do you think there is need for better evidence to determine the feasibility and safety of the MI combined approach?
   1. Yes
   2. No
