# Supplementary figures and images for: Global survey on the surgical management of patients affected by colorectal cancer with synchronous liver metastases: impact of surgical specialty and geographic region
Source: Surg Endosc. 2023 Mar 6;37(6):4658–72. doi: 10.1007/s00464-023-09917-8 (PMC10234876; doi:10.1007/s00464-023-09917-8)

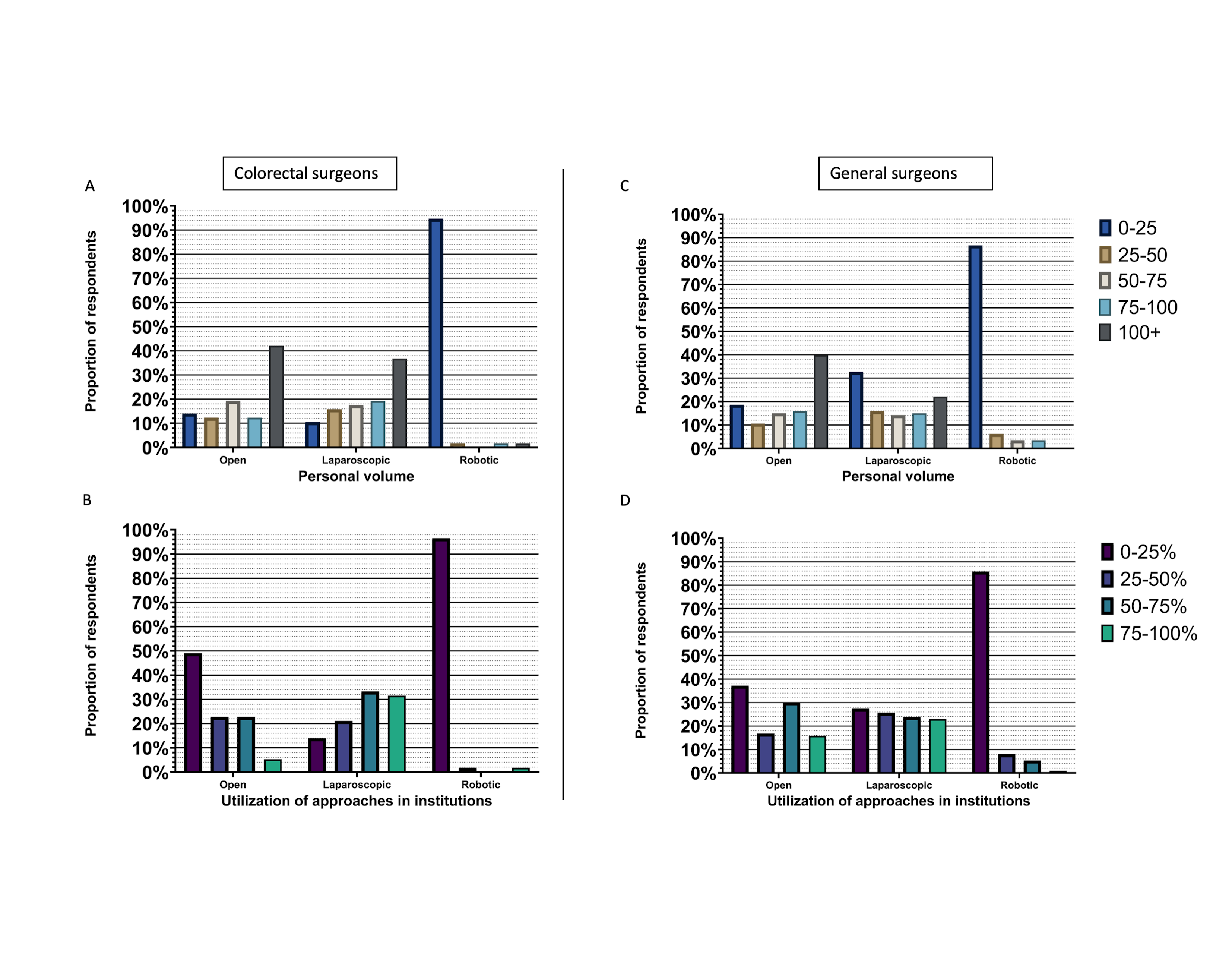

Supplement: Supplementary file 4 — Supplementary file4 (TIFF 33592 kb)—Supplementary figure 2. Volume and institutional usage of the different surgical approaches for colon resections. A) Personal volume of colorectal surgeons B) Utilized surgical approaches in colorectal surgeons’ institutions C) Personal volume of general surgeons D) Utilized surgical approaches in general surgeons’ institutions [file 464_2023_9917_MOESM4_ESM.tiff]

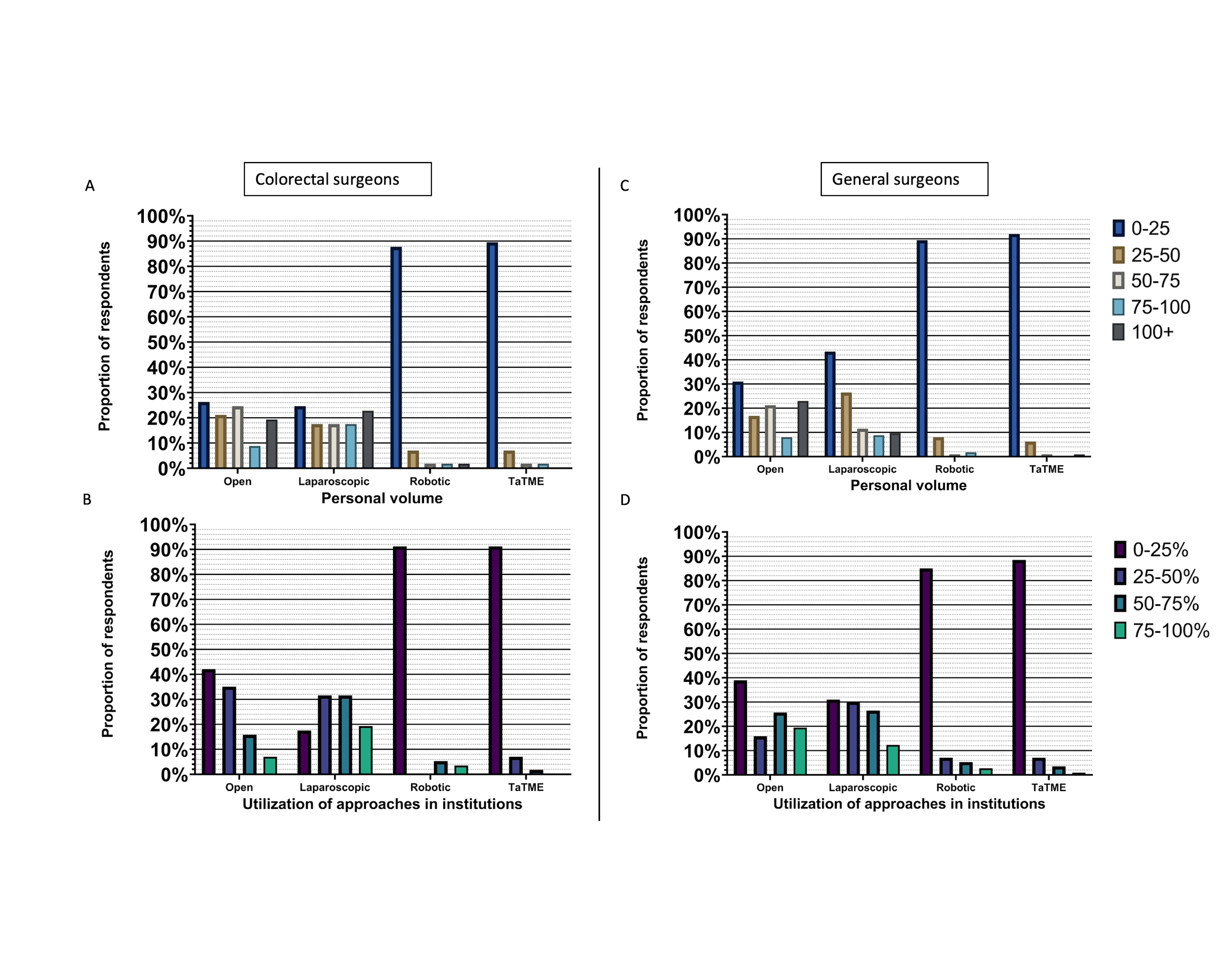

Supplement: Supplementary file 5 — Supplementary file5 (TIFF 33566 kb)—Supplementary figure 3. Volume and institutional usage of the different surgical approaches for rectal resections. A) Personal volume of colorectal surgeons B) Utilized approaches in colorectal surgeons’ institutions C) Personal volume of general surgeons D) Utilized approaches in general surgeons’ institutions [file 464_2023_9917_MOESM5_ESM.tiff]

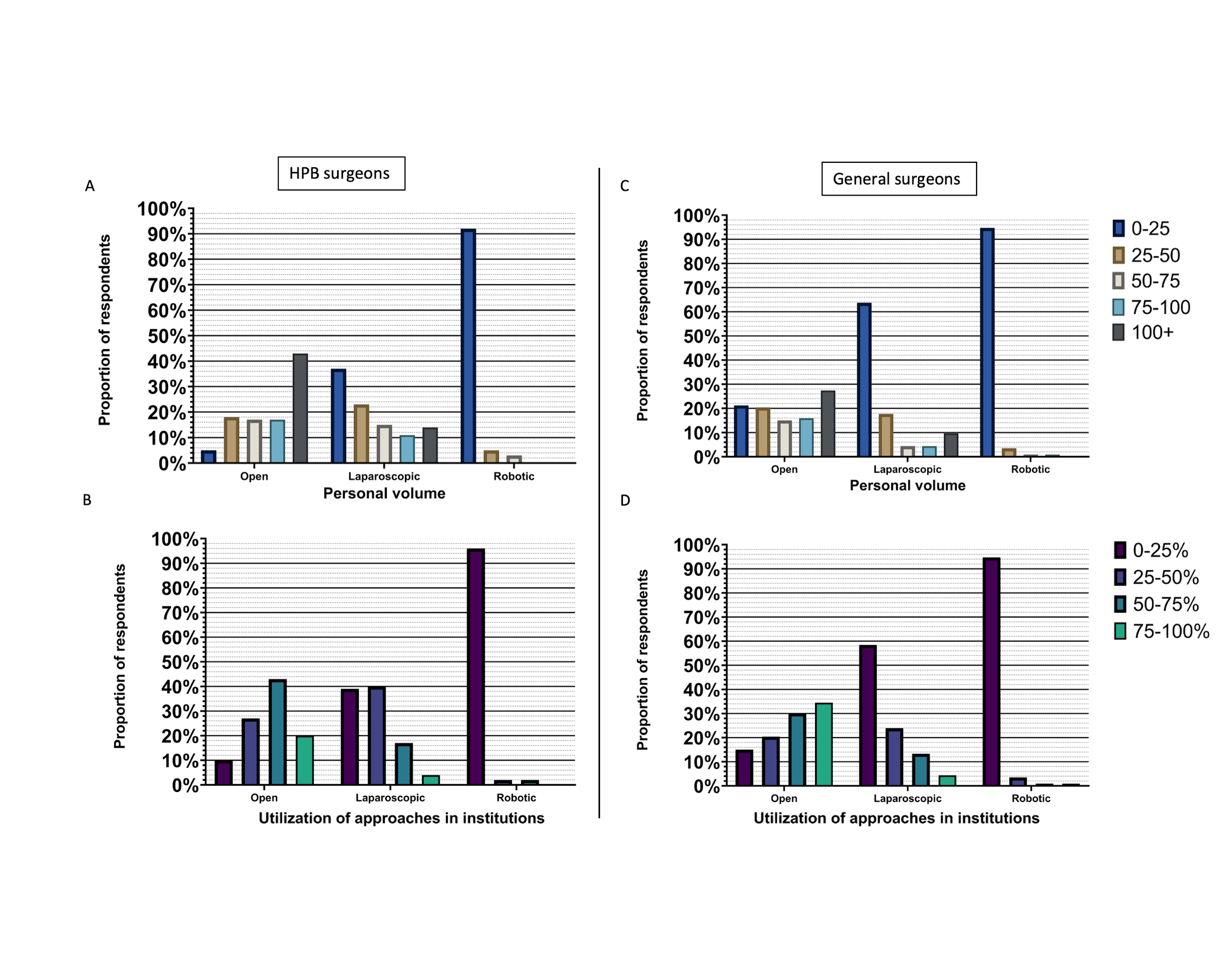

Supplement: Supplementary file 6 — Supplementary file6 (TIFF 33592 kb)—Supplementary figure 4. Volume and institutional usage of the different surgical approaches for liver resections. A) Personal volume of HPB surgeons B) Utilized surgical approaches in HPB surgeons’ institutions C) Personal volume of general surgeons D) Utilized surgical approaches in general surgeons’ institutions [file 464_2023_9917_MOESM6_ESM.tiff]

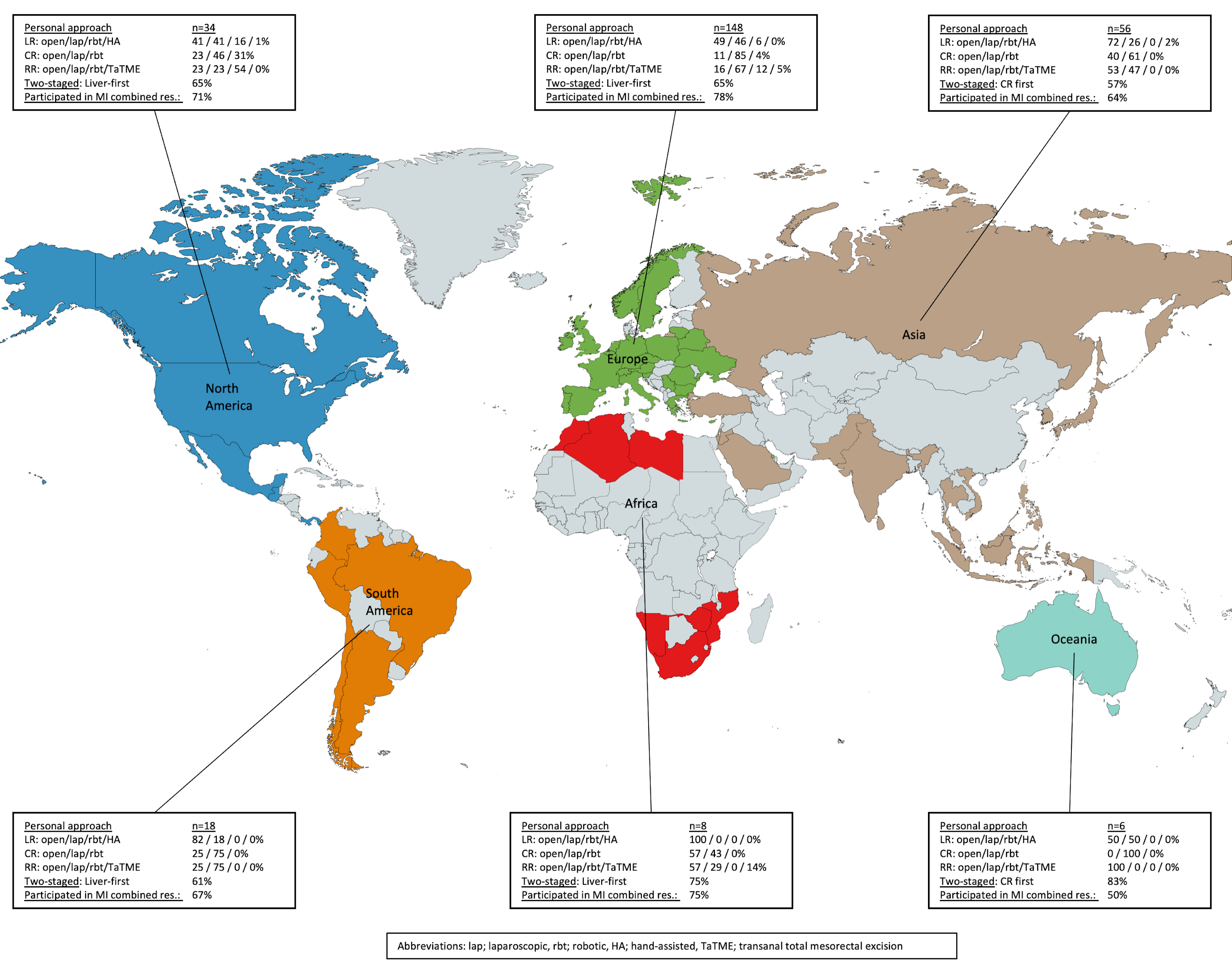

Supplement: Supplementary file 7 — Supplementary file7 (TIFF 33592 kb)—Supplementary figure 5. Countries of residence of respondents and intercontinental differences in used approaches and strategies in patients with sCRLM [file 464_2023_9917_MOESM7_ESM.tiff]

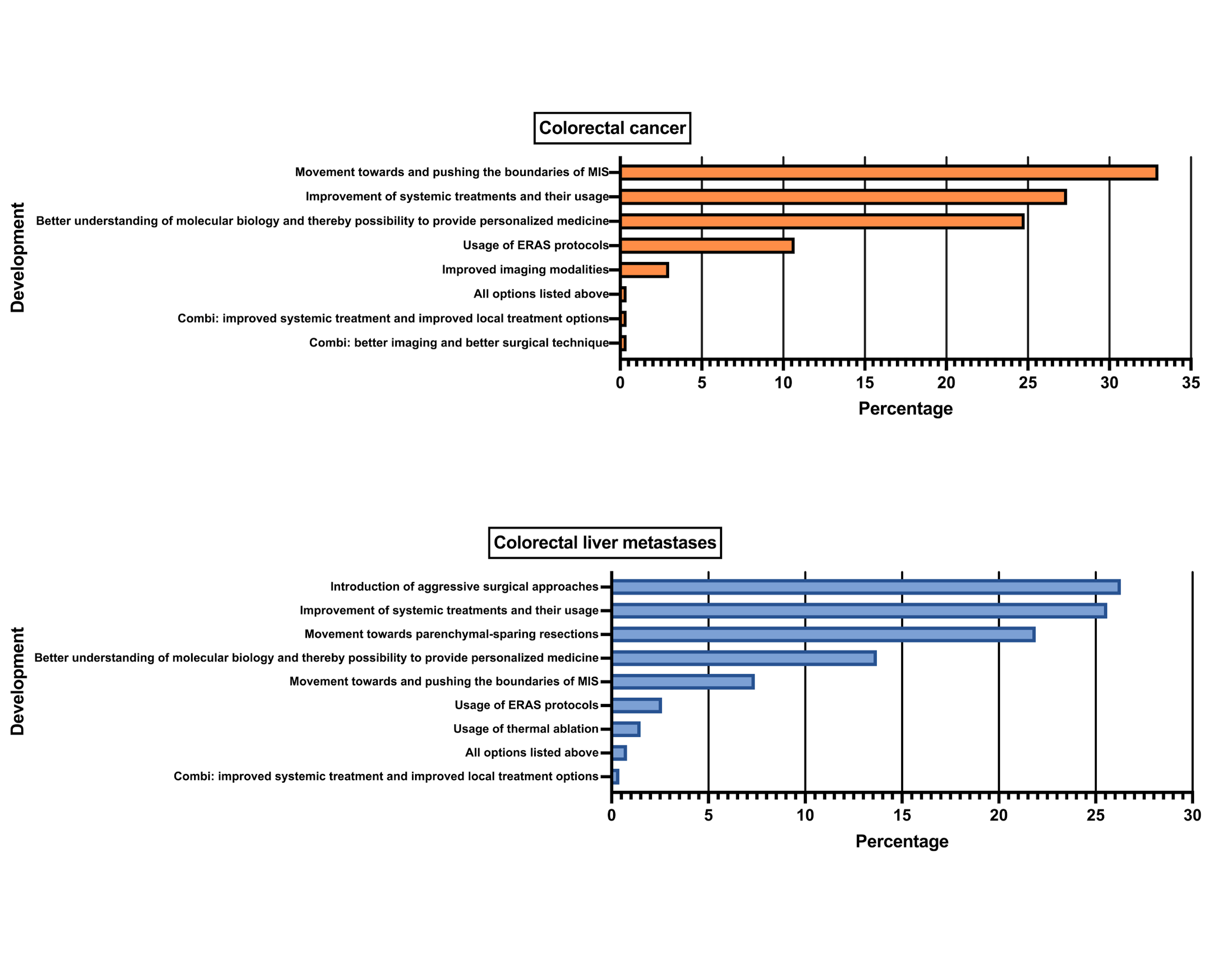

Supplement: Supplementary file 8 — Supplementary file6 (TIFF 33592 kb)—Supplementary figure 6. Viewpoints of respondents on the developments in the treatment of colorectal cancer and colorectal liver metastases which made the most clinical impact over the last two decades [file 464_2023_9917_MOESM8_ESM.tiff]
